# Evaluating Staffing Guidelines Using Trauma Volume by Season, Day, and Time of Day at a Level 1 Trauma Center in Rural Appalachia

**DOI:** 10.7759/cureus.61429

**Published:** 2024-05-31

**Authors:** Viraj V Brahmbhatt, Matthew Leonard, Bracken Burns

**Affiliations:** 1 Trauma, Union College/Albany Medical College, Albany, USA; 2 Trauma, Ballad Health Trauma Services, Johnson City, USA; 3 Surgery, East Tennessee State University, Johnson City, USA

**Keywords:** work schedule, motor vehicle injury, emergency medicine and trauma, quality improvement projects, medical staffing

## Abstract

Background

Trauma is regarded as randomly occurring, but patterns exist for trauma volume which are useful in staffing guidelines and resource allocation. Literature on trauma admissions volume has been centered around geographically/climatically diverse centers and has often not considered many different temporal factors at once. Additionally, studies on trauma volume and staffing centered around rural or southern Trauma 1 centers were largely absent in the literature. Based on this, a study on our Trauma 1 center was deemed appropriate.

Objective

The objective of this study was to determine significant trends in trauma admissions and use this information to assess current staffing. This assessment was conducted through a retrospective analysis of patients admitted to the emergency department at our center.

Methodology

The retrospective data analysis study was conducted using data obtained locally and then subsequently uploaded onto the National Trauma Data Bank (NTDB). Patients included all trauma activations and consults to the trauma team above the age of 18. We analyzed the data by season, day, and time of admission to identify trends. Chi-square analysis was used to establish significance in comparing groups (day of the week, hour of day, and season of the year). Factors such as Injury Severity Score (ISS), Glasgow Coma Scale (GCS), and length of stay (hospital days) were used to determine patterns of trauma severity.

Results

A total of 15,418 patients (8,307 males, 7,111 females) were analyzed in the dataset. The mean ISS was 8.14, and the mean GCS was 14.22. Weekends had significantly greater trauma volume than weekdays (*P *< 0.05). Motor vehicle collision (MVC), motorcycle, bicycle, and all-terrain vehicle (ATV) traumas were all significantly greater in summer. Bicycle and ATV trauma were significantly lowest in winter (*P *< 0.05). Admissions began to rise at 7 am and peaked at 5 pm. ISS, GCS, and hospital days did not significantly differ based on all groups assessed. Variation in trauma peak time across days of the week was insignificant. Our study discovered key findings in the form of increased trauma patient volume in summer, weekends, and between the hours of 1 and 9 pm. Our project found that the current staffing presents a mismatch in terms of addressing periods of high trauma. Staffing is lower on weekends, and there are conflicting administrative tasks scheduled during peak trauma hours (1-7 pm). This could be addressed by adjusting shift schedules to align better with periods of high trauma, such as increasing the workforce on weekends and decreasing it on weekdays. During summer months, adding additional float shift staff may help to better address the peak trauma volume.

Conclusions

Currently, there is evidence to suggest that high trauma times correspond with times of low staffing. Based on our study, there was evidence to show that low staffing periods corresponded with high trauma times. This volume-to-staffing mismatch may contribute to underlying problems such as long wait times, overworked providers, and ED overcrowding. Future studies may choose to focus on quality differences and wait times, aiming to quantify the effect of the mismatch of resources on patient volume.

## Introduction

Implementing reforms to trauma systems to boost patient experience and workflow has been the focus of many interventions [1-3]. Trauma remains one of the leading causes of death and injury across different age and demographic groups [4-6]. Based on this, improvements aimed at innovating the trauma department may lead to improvements in medical delivery across a large population. The volume of trauma cases can be addressed by implementing strategies to mitigate factors contributing to trauma or by improving the organization of the trauma department. 

Previous studies have shown that trauma volume varies depending on the time of year and the type of trauma [6-8]. Identifying these high-trauma times may be useful in structuring staffing and resources. Existing studies on the patterns in trauma occurrence have chosen to identify trends at their respective medical center. Based on the recommendations published by the American Association for the Surgery of Trauma, recommendations are unique to each center, and the application of results to a unique environment may not provide accurate information. This encourages the replication and justifies the differing results seen in the literature.

The existing studies investigating temporal trends in trauma admissions come from a variety of different locations as well as different trauma center levels. Studies from a variety of regions have chosen to investigate seasonal trends. Summer months were found to have greater rates of trauma, though not all studies found the greater rates to be significantly different [9-11]. Day of the week was another important report, with studies finding weekends with greater admissions and often greater injury severity as well [6,12,13]. Studies have also found that trauma admissions are higher in the afternoons and evenings [14,15]. Based on these considerations, this study examined variations in season, day of the week, and time of day. The current literature is unclear on whether the temporal trends are consistent across diverse trauma centers. The results from this study aim to bridge this gap.

The existing literature suggests general patterns that may apply; however, the studies were not based in Appalachia. The unique climate, geography, and patient demographics of the area may provide a unique insight into resource allocation in the area. This study aims to provide insight into times of high trauma admissions to help determine staffing guidelines. Restructuring staffing and resource allocation can help to address high patient volume during *peak hours* and reduce excess staffing during low-volume times. This can enhance patient experience while simultaneously reducing operational costs.

Guidelines that aim to better manage trauma volume may improve outcomes as well. The literature has shown mixed results on whether increased volume correlates to differences in outcomes. Depending on sample size and case mix, the effect of volume may be different [16-19]. This suggests that though trauma volume may not be a direct indicator of patient mortality, better management of trauma volume may lead to better outcomes. This study does not aim to investigate patient outcomes but instead looks into significant distributions and trends of trauma volume and the corresponding staffing. Many hospitals are operating in the southern United States using a system similar to that of our institution. For these hospitals, objective information on admission trends may be useful to build future staffing and resource allocation guidelines. Colloquially, workers in the ED often refer to certain periods as *busy times*, but objectively identifying these periods is an important step going forward. This study aims to bridge this gap and to contribute an objective picture of trauma volume fluctuations. The goal of this analysis is to form a baseline that may be useful for our hospital and similar hospitals operating in a similar climate and operational structure. 

Given the temperate climate and the young median age, we anticipate less variation in trauma attributed to falls [20]. Additionally, activities such as motorcycling and the use of all-terrain vehicles (ATVs) are prevalent in the area and are likely to constitute a large portion of injuries [21]. Using the results from this study, this center as well as other centers operating in a similar climate can modify their staffing and resources to better address patient volume and demands.

The abstract was presented as a quick shot oral presentation at the South Eastern Surgical Conference.

## Materials and methods

This study obtained Institutional Review Board clearance from the Ballad Health System Institutional Board 000032043 before commencement. All data came from a Level 1 Trauma Center in East Tennessee. Data were obtained from the center’s trauma registry. The data included patients aged 18 and above who were admitted for trauma-related injuries between January 1, 2017, and December 31, 2022. The data were obtained locally and then subsequently uploaded onto the National Trauma Data Bank (NTDB). Patient data included age, sex, time of admission, date of admission, Injury Severity Score (ISS), Glasgow Coma Scale (GCS), hospital days (LOS), intensive care unit days (ICU), discharge status, injury type, and injury mechanism. Data is entered manually by trauma registrars. The facility is required to employ 1 trauma registrar per 500-750 admissions. All registrars meet state requirements, which include continuous training including 8 educational hours per year, attendance of regular meetings, and Trauma Quality Improvement Program (TQIP) annual meetings. All patients included in the study were evaluated in the Emergency Department by the trauma team. This included all trauma activations and consults with the trauma team from the emergency department. The trauma activation criteria are shown in the Appendix. All patients met the NTDB inclusion criteria.

Trauma volume was separated by type and plotted by month to identify and depict patterns of high-trauma months. Data were analyzed to determine trends and characteristics of trauma admissions by time. Each trauma entry was coded as season based on the definitions of summer (June 21-September 23), spring (March 21-June 21), autumn (September 23-December 22), and winter (December 21-March 21). Summary statistics were calculated based on demographic factors such as age, sex distribution, trauma type distribution, ISS, and GCS. Trauma volume was assessed using chi-squared analysis using the mean as the value of comparison. The choice to use hypothesis testing rather than simply employing descriptive results was based on a thorough literature analysis. Papers analyzing temporal trauma volume had chosen to utilize similar statistical methods, and it was determined that these methods would be appropriate for this study as well. The selection of a post hoc test was done to determine what groups were significant. Based on the literature, chi-squared analysis was chosen. ISS, hospital days, and GCS in each season were assessed through analysis of variance (ANOVA) to determine the significance of the difference. Following this, post hoc testing using Tukey-Kramer Honestly Significant Difference (HSD) was used to identify unique groups. The analysis looked at the data based on season, day of the week, and hour of admission. Microsoft Excel 2023 and JMP PRO16 were utilized for data query and analysis.

## Results

A total of 15,418 patients (8,307 males and 7,111 females) were analyzed. The mean ISS was 8.14, and the mean GCS was 14.22. The most common injury type was falls, which accounted for 8,219 (53.31% of all traumas in the dataset). In the study, the majority of traumas were blunt, accounting for 13,999 cases (90.8%), followed by 1,079 cases (7%) classified as penetrating, 246 cases (1.6%) categorized as burn injuries, and the remaining 94 cases (0.6%) classified as Other or NA.

Analysis based on season

**Figure 1:**
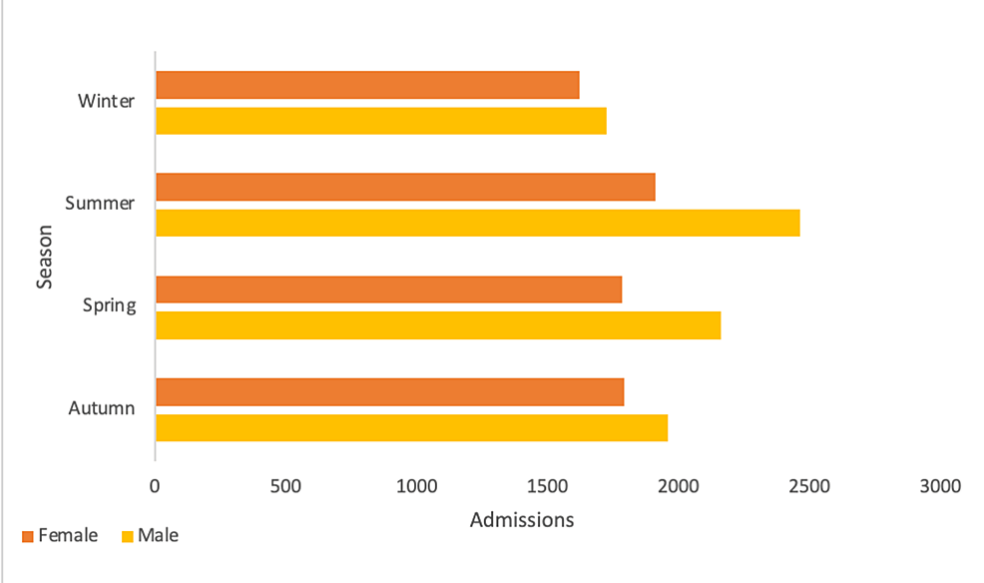
Trauma admissions: male admissions greater in all seasons; admissions are greatest in both genders during summer (P < 0.05).

Summer had the greatest trauma volume followed by spring, autumn, and winter (*P *< 0.0001), as shown in Figure 1. Adjusted residual testing revealed that females in winter and males in summer had significantly higher values than the mean, whereas females in summer and spring, as well as males in winter, had values significantly lower than the mean (*P* < 0.05).

**Figure 2:**
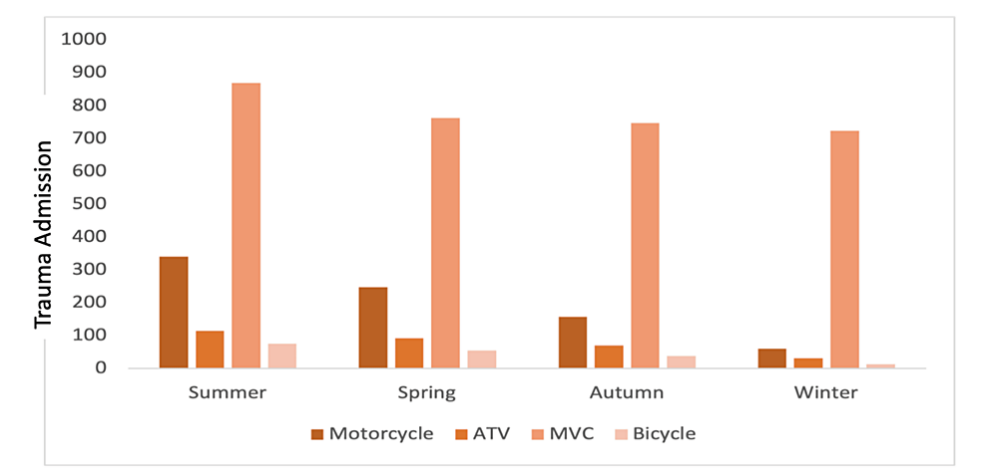
Trauma shown for vehicles, sorted by season. Each season presented unique patterns for motorcycle, bicycle, and ATV traumas, with distinct occurrences observed in both winter and summer. MVC trauma was significantly highest in summer (*P *< 0.05). MVC refers to motor vehicle, while ATV refers to all-terrain vehicle.

There was variation in all vehicle-related trauma. ATV accidents had the greatest variation, while MVC had the least variation. Trauma volume for vehicular accidents displayed greatest rates in the summer. There was variation in significance among types of vehicular accidents. Each season was significantly unique for motorcycle accidents (*P *< 0.05). Bicycle and ATV trauma were statistically greatest in summer and lowest in winter, as compared to autumn and spring (*P *< 0.05). There was no difference between spring and autumn. MVC trauma was significantly greater in summer than the other three seasons, which did not differ (*P *< 0.05) (Figure 2). Average ISS by vehicular trauma was assessed with motorcycle having the greatest average ISS at 10.6. However, the variation observed in the average ISS was found to be insignificant (*P* = 0.757). ISS and GCS showed no significant variation among seasons. Autumn had an average ISS of 8.4 (95% confidence interval [CI] 8.17-8.62) and an average GCS of 14.2 (95% CI 14.12-14.29). Spring had an average ISS of 8.1 (95% CI 7.91-8.37) and an average GCS of 14.2 (95% CI 14.13-14.30). Summer had an average ISS of 8.1 (95% CI 7.86-8.27) and an average GCS of 14.2 (95% CI 14.16-14.31). Winter had an average ISS of 8.0 (95% CI 7.73-8.18) and an average GCS of 14.2 (95% CI 14.12-14.30).

Analysis based on day of the week

**Figure 3:**
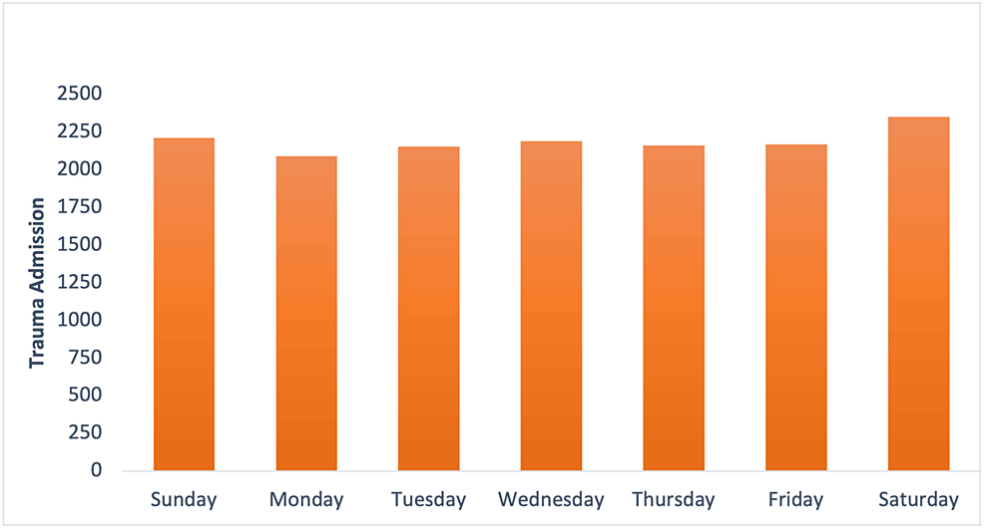
Trauma sorted by day of the week. Trauma peaks during the weekend, with Saturdays having the greatest trauma rates. Weekends had greater trauma rates compared to the work week (*P *< 0.05).

Trauma rates were greater on the weekends than during weekdays, and this difference was statistically significant (*P *= 0.036), as shown in Figure 3. The difference seen in ISS, GCS, and hospital days between weekends and weekdays were not significant (*P *= 0.932, *P *= 0.997, and *P *= 0.906). The hospital days variation was insignificant (*P *= 0.999). Overall, the complexity in patients (as seen through ISS, GCS, and length of stay) did not vary significantly across days of the week. The mean and median time of admission was approximately 2 pm. This represented the peak admission time. The analysis across days of the week showed that the distribution pattern in admissions remained constant across days of the week. The only discrepancy that could be seen was on Saturday and Sunday where the mean and the median were slightly earlier than during the week. 

Analysis based on the time of admission

**Figure 4:**
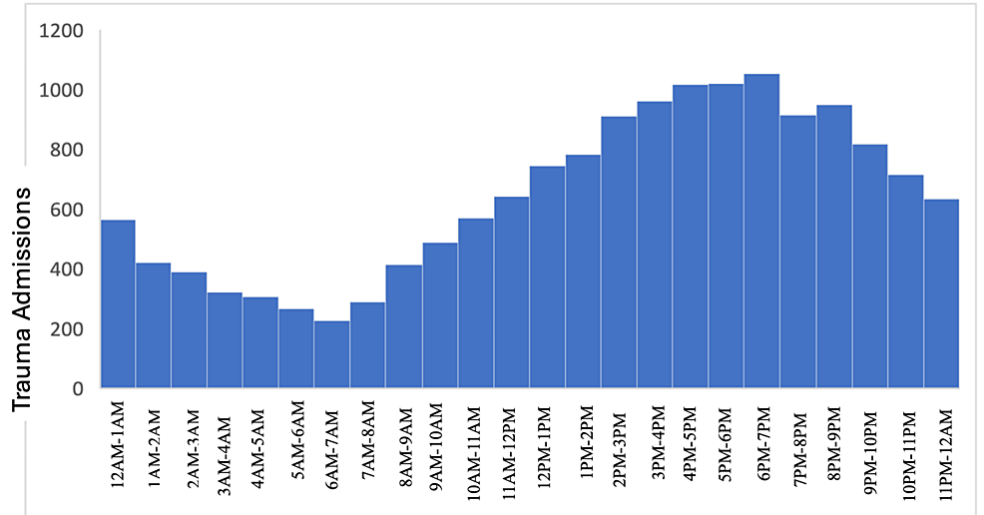
Time distribution of admissions. Admissions began to rise at 7 am and peaked at 5 pm. The admissions pattern may be used to guide staffing decisions. The graph shows frequency of presentation to the Emergency Department separated into frequency category by hour.

**Figure 5:**
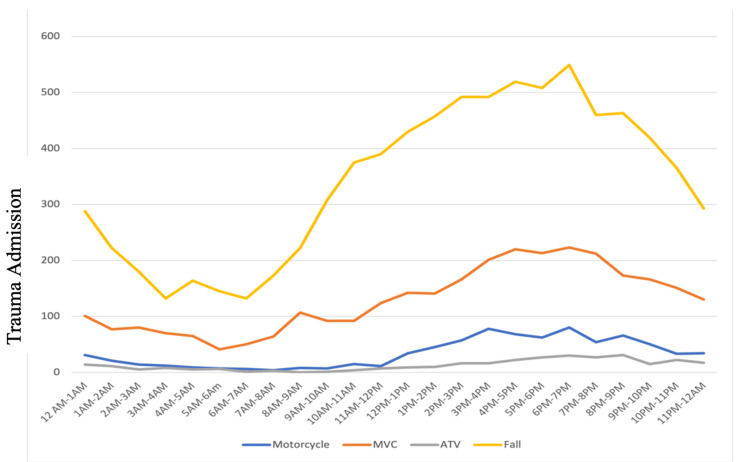
Trauma admissions for four major categories plotted by hour of day. Falls account for most of the trauma admissions and peaks later in the day. MVC refers to motor vehicle, while ATV refers to all-terrain vehicle. The frequency of admission is shown sorted by hour of the day.

The average time of arrival across all months was approximately 2 pm. The greatest admissions occurred between 3 and 6 pm. Overnight (11 pm-4 am), admissions remained low and relatively stable (Figure 4). Across different types of trauma, the pattern remained the same. This seems to indicate that there is a *peak trauma time* across all admissions and staffing can be based around these timelines. Trauma admissions by trauma type peaked at different times (Figure 5), although the trend was similar across different trauma types. Falls peaked later than vehicle-based traumas. Falls also accounted for majority of traumas at the center. Trauma admissions remained low overnight across all categories (Figure 5). Variation shown in the ISS, GCS, and hospital days by hour of admission was not significant. The level of each remained relatively stable. There were small, insignificant fluctuations. The key takeaway here seems to indicate that the severity of the trauma does not vary across periods of time.

## Discussion

Although the occurrence of trauma and trauma admissions are independent, chance events, certain variables have been identified as significant predictors of trauma rates and may be effective in predictive guidelines. All of these factors can help drive administrative decision-making and prepare for predictable trauma volume. One potential implication of this is rescheduling administrative tasks that occur during times of high trauma volume. The conclusions drawn from seasonal trauma trends point towards higher trauma volume during summer months. This pattern of trauma levels reaching a peak during summer months was found in other studies as well [6, 9,10,11,22]. An earlier study based in western Massachusetts displayed a unique pattern from what was seen in our study. This was specifically true in seasonal variation and variation in types of traumas. The Massachusetts study found greater falls in the winter, and the lowest MCC (motorcycle) trauma in the winter [22]. An international study based in Germany found unique patterns in the literature. The findings from this correspond with the findings from our study in terms of increased trauma rates during the summer months and late afternoon hours [10]. Compared to the existing literature on variation across days of the week, our results agreed with the results that weekends had significantly greater trauma volume; however, the trauma severity on weekends was not different [6,12,13]. The results from our study also correspond with findings from existing literature which found greater trauma rates during afternoons and evenings [14, 15].

Seasonal differences in trauma are less likely to vary due to the temperate climate of East Tennessee [23]. However, warm seasons are more likely to see an increase in the use of ATVs and Motorcycles [24]. This finding from the past literature was confirmed by the data in our study as well. The increased incidence of all types of traumas during the summer months may be due to increased activity. During the summer and warmer months, individuals are more likely to spend time outside or participate in physical activities [7,25]. This increase in activity likely underlines the increased incidence of trauma. MVC-based trauma was the least volatile among the vehicle-based trauma types assessed. It was also the highest in vehicle-based trauma. This is likely due to automobiles being the primary form of transportation. The higher volume of automobiles leads to a higher likelihood of a crash occurring during the use of automobiles. Other vehicle trauma types had a significant decrease during winter; however, MVC did not show this drop. This finding agreed with findings from other centers [22]. Based on this, the arrival of MVC is not likely to show great variation, regardless of the location of the center. 

This pattern of higher trauma in warm months also continued into falls. The assumption is that fall-based trauma is likely greater in winter months due to ice and snow [22]. The data suggests that falls were significantly greater in the summer and autumn (p=0.021). Chi-square analysis confirmed that the variation observed in fall rates was significantly different. The assumption that falls are greater in the winter is based on the observation that ice due to low temperatures only forms during winter months. However, in southern Appalachia, the temperate winters may not have significant winter months. As a result, the fall rates do not dramatically change during this time. The greater fall rates during the summer months may also be explained due to increased activity during this time. During this time, citizens may engage in behaviors and activities that increase their risk of falling. Studies finding greater falls during winter months typically centered in areas with more extreme winter weather. As a result, the increase in falls during winter may be attributed to weather conditions, while the high falls during summer in our study may be attributed to changes in behavior associated with seasonality [22].

The analysis based on days of the week revealed greater rates during the weekend. This finding corresponds with the other studies as well [6]. This finding may be relevant for staffing guidelines as institutions tend to reduce staffing during the weekend. Current literature has not investigated the association between staffing and patient outcomes; however, this study has found a reverse association between staffing and trauma volume. Though the results from a single center may not apply to all hospitals, this study was conducted on a large patient dataset from a Trauma 1 center. Given the large number of southern hospitals and few publications centered around these studies, this study presents findings that may be particularly relevant for a large subset of American hospitals. In past studies, it has been found that staffing and quality patient care often change based on the day of the week (and other shift schedules) [26]. Though this study did not investigate patient outcomes directly, a clear mismatch of staffing resources and trauma admission volume was observed. Based on this, the fluctuations in quality care seen in other studies may be correlated to understaffing during times of high trauma. Future studies may consider incorporating all three points together in a large patient population to attempt to bridge the connection between admission volume and quality care. 

The findings suggest that current staffing is not matched with trauma volume. Based on this, maintaining or increasing staffing may be a positive strategy to address trauma volume. The injury severity based on ISS, GCS, and Hospital days did not differ among the days. This finding was unique from many other studies. This finding does not provide additional information to drive staffing. There was a pattern of average hospital days peaking on Wednesdays; however, this peak was not significant. 

The analysis based on time of day revealed a peak in trauma admission between 3 and 6 pm. The median and mean admission time was around 2 pm and remained the same across all days of the week. This stability in admissions was unique from many other publications. In other studies, admissions typically peaked later in the evening on weekends. This study found slightly earlier admissions during the weekend, but the discrepancy was not significant. The ISS, GCS, and hospital days varied throughout the day, but the observed variation was not significant. The trauma type peaked at different times in the afternoon. Falls peaked at the latest compared to other trauma types.

Based on this model and the trends in trauma volume observed, a more clear relationship between the two may be seen. The current staffing includes physicians specialized in trauma surgery, advanced practice extenders, and resident physicians. The facility has different staffing based on the day of the week and the time of the day. During the weekdays, the trauma center staffing is done through one physician staffing the facility at all hours. In addition to this, there are four physician extenders, and resident physicians (who are responsible for services beyond trauma alone). Overnight, there is one physician and three resident physicians available. During weekends, there is always one physician available, three physician extenders, and three resident physicians (who are responsible for services beyond trauma alone). The staffing going into the weekend changes as typically two other physicians are covering other services for which trauma physicians are responsible, but on the weekends, there is only one other physician available. Additionally, physicians work based on 12-hour shifts during the week, but on the weekends the physicians work 24-hour shifts. Using the full-time equivalent (FTE) definition of 40 hours/week, all discussed staff members are FTE for the system. The comparison of trauma volume to staff availability involves the investigation of several staffing members present at any given point rather than the total number of FTEs employed by the practice. As such, the FTE definition model was not used. Rather, the discussion presents the idea of a drop-off in staffing corresponding to periods of increased trauma volume, such as during the weekends. 

The general pattern seen is a reduction in staffing overnight as well as during the weekend. This staffing pattern is justified based on the lifestyle habit of preferring time off during the weekends. However, this staffing does not reflect the pattern seen in trauma admissions. Trauma rates were greatest during the weekend. Additionally, the resident physicians often schedule meetings and educational seminars between the hours of 12-5, which corresponds to the period when trauma rates increase. Given that trauma volume significantly differs by time of day, day, and season, the results of the study recommend future staffing decisions should consider these patterns. This study did not investigate whether the mismatch was linked to a significant change in patient outcomes; however, future studies may consider this approach. The results from these future studies will provide further recommendations for staffing practices. 

Based on the trends seen in trauma admissions, some recommendations may help to remediate the mismatch between admissions volume and staff scheduling. Meetings would be better held either before 1 pm or after 9 pm as these are the periods with lower trauma volume. This recommendation is based off of the assumption that these times would have all personnel at the hospital. Additionally, these times are periods of lower trauma as compared to the period between 1 pm and 5 pm, which is typically when meetings/seminars are currently held. Currently, meetings and administrative tasks may be held during this time (between 12 and 5), which would result in staff being taken off of the ED floor when trauma volume is increasing. This is based on the results of this study which shows increasing trauma volume between the hours of 12-5.

Another interesting aspect is that the physician shift changes at 5 pm during the weekdays. This period is when the greatest trauma rates are. It may be better advised to have a shift overlap during this time to better address the higher rates. Additionally, the staffing patterns may be changed based on the season. During the winter, staffing may be lightened as trauma volume is significantly lower. During the summer, however, there is greater trauma volume, which should warrant increased staffing. Since it is difficult to staff for a single season alone, this may warrant more creative staffing practices. For example, the practice may opt to schedule more float shifts (i.e., having more physicians on call to better serve the peak trauma times). These float shifts could also be extended to physician extenders and resident physicians. An extreme and expensive option would be the use of locum tenens, which would be less favorable due to the notable drawbacks of this approach. 

This study displayed interesting findings about trends in trauma admissions at a Level 1 trauma center in southern Appalachia. The study’s findings about increased trauma volume during weekends are relevant for structuring staffing guidelines. In addition to this, the hours of peak trauma (1 pm-7 pm) are the times of shift changes, administrative tasks, etc. Given the results of the study, administrative tasks are best to be scheduled before noon. Additionally, it may be a better option to schedule an overlapping shift between 3 and 6, so that there are additional workers present at the peak trauma time. For example, the first shift may end at 7, while the second shift may begin at 3. Through this, there are increased chances of better handoff of information between shifts and increased staffing during peak trauma times. 

It may be beneficial to determine whether there are differences in patient outcomes based on factors such as time of day, season, and day of the week. It may be beneficial to determine periods when trauma outcomes are worse and to adequately address this discrepancy. Future studies may also choose to investigate trauma referrals to determine when to increase staffing in common referral areas such as orthopedic trauma. Ultimately, large changes in staffing behaviors are difficult to act upon and not all changes may be possible. Simple changes such as moving administrative tasks before noon may be a strong starting point for hospitals. Further changes may focus on moving shift start and end times. The most difficult of changes may involve asking workers to work longer hours, weekends, or longer during the summer months. This is due to the lifestyle-based staffing practices currently in place. Currently, staffing is based on maximizing lifestyle. For example, staff would prefer to work on weekdays and take weekends off. Additionally, it is idealistic to finish the workday before 8 pm. This pattern is not always true across all types of staffing in the emergency department. For example, physicians are scheduled on 24-hour shifts. However, these shifts are not often scheduled regularly (every week) and are typically spaced out to provide a compromise between extended working hours and lifestyle preferences. 

The study design involved a large dataset, but the data was limited to a single center. Similar to the other studies in the existing literature, many of the findings are only relevant to medical centers operating in a similar area. This study presented a unique view of the mismatch which was absent from existing literature. Given that not all medical centers employ the same staffing model, this finding may be limited. Additionally, the setting of Appalachia is very unique, and findings from this setting are limited to medical centers operating within this unique area. Additionally, implementation of the findings is very limited. Though the principle of matching staffing with demand seems reasonable, it may become difficult in practice. Additionally, as with other studies, limitations of the results stem from climate and staffing differences across centers. For example, centers located far from the region of the Southern Appalachia may not find the results to be applicable. Additionally, hospitals in other regions may employ a different staffing model. Our hospital includes a training/educational component and this presents itself in the staffing. Hospitals that do not include an educational component may find significant differences in staffing. Within traumatology, there has long been the mention of a *trauma season* and certain hours of the day are often regarded as *busy shifts*. However, a quantitative assessment of these patterns offers an objective insight into the trends of trauma volume. Using this objective data, staffing and resource allocation decisions may be better informed in our health system and similar systems across the United States.

## Conclusions

The results of the study are that trauma rates significantly vary, and the current staffing model used is not made to match these fluctuations. Based on the findings of this study, future staffing models should consider matching the trends in trauma volume. By better aligning staffing with trauma arrivals, there may be improvements to the ED workflow and patient experience. Determining the significance of these changes may be the objective of future research.

## Figures and Tables

**Figure 6:**
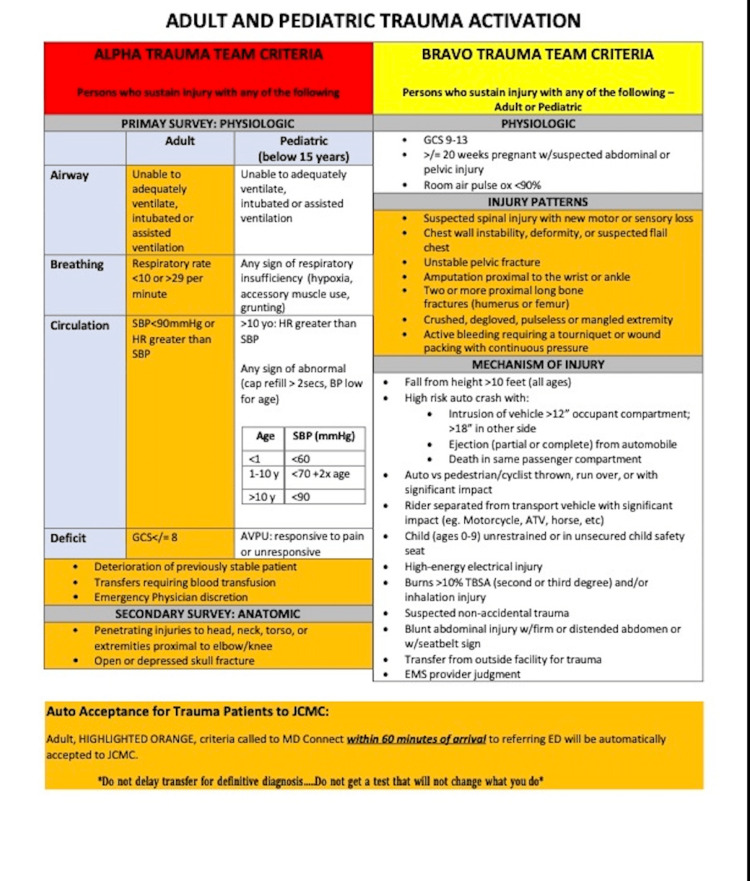
Trauma activation criteria to provide more information on the information referenced in the Materials section.

## Appendices

Appendix

## References

[1] Mullins RJ (1999). A historical perspective of trauma system development in the United States. J Trauma.

[2] Mwandri M, Stewart B, Hardcastle TC, Rubiano AM, Gruen RL (2017). Organised trauma systems and designated trauma centres for improving outcomes in injured patients. Cochrane Database Syst Rev.

[3] Evans DC, Andrusiek DL, Sobolev B (2013). Process Mapping of a Regional Trauma System. Patient Flow: Reducing Delay in Healthcare Delivery. International Series in Operations Research & Management Science.

[4] Heron M (2021). Deaths: leading causes for 2018. national center for health statistics.

[5] Scholl L, Seth P, Kariisa M, Wilson N, Baldwin G (2018). Drug and opioid-involved overdose deaths — united states, 2013-2017. MMWR Morb Mortal Wkly Rep.

[6] Stonko DP, Dennis BM, Callcut RA, Betzold RD, Smith MC, Medvecz AJ, Guillamondegui OD (2018). Identifying temporal patterns in trauma admissions: Informing resource allocation. PLoS One.

[7] Pivarnik JM, Reeves MJ, Rafferty AP (2003). Seasonal variation in adult leisure-time physical activity. Med Sci Sports Exerc.

[8] Craig J, Hinds JD, Kealey DW, Heyes GJ (2014). The burden of motorcycle trauma and seasonal change at a regional trauma centre. Ulster Med J.

[9] Rising WR, O'Daniel JA, Roberts CS (2006). Correlating weather and trauma admissions at a level I trauma center. J Trauma.

[10] Pape-Köhler CI, Simanski C, Nienaber U, Lefering R (2014). External factors and the incidence of severe trauma: time, date, season and moon. Injury.

[11] Wilson JM, Staley CA, Boden AL, Boissonneault AR, Schwartz AM, Schenker ML (2018). The effect of season and weather on orthopaedic trauma: consult volume is significantly correlated with daily weather. Adv Orthop.

[12] Friede KA, Osborne MC, Erickson DJ (2009). Predicting trauma admissions: the effect of weather, weekday, and other variables. Minn Med.

[13] Bhattacharyya T, Millham FH (2001). Relationship between weather and seasonal factors and trauma admission volume at a level I trauma center. J Trauma.

[14] Vaziri K, Roland JC, Robinson L, Fakhry SM (2023). Optimizing physician staffing and resource allocation: sine-wave variation in hourly trauma admission volume. J Trauma.

[15] Kieffer WK, Michalik DV, Gallagher K, McFadyen I, Bernard J, Rogers BA (2016). Temporal variation in major trauma admissions. Ann R Coll Surg Engl.

[16] London JA, Battistella FD (2003). Is there a relationship between trauma center volume and mortality?. J Trauma.

[17] Glance LG, Osler TM, Dick A, Mukamel D (2004). The relation between trauma center outcome and volume in the National Trauma Databank. J Trauma.

[18] Caputo LM, Salottolo KM, Slone DS, Mains CW, Bar-Or D (2014). The relationship between patient volume and mortality in American trauma centres: a systematic review of the evidence. Injury.

[19] Calland JF, Stukenborg GJ (2016). Trauma centre patient volume and inpatient mortality risk reconsidered. Injury.

[20] (2023). Johnson City. https://datacommons.org/place/geoId/4738320/.

[21] Testerman GM (2009). 300 all-terrain vehicle crashes: an East Tennessee trauma center's experience. Tenn Med.

[22] Nahmias J, Poola S, Doben A, Garb J, Gross RI (2017). Seasonal variation of trauma in western Massachusetts: fact or folklore. Trauma Surg Acute Care Open.

[23] (2023). Tennessee Climatology. https://www.etsu.edu/cas/geosciences/tn-climate/tn-climatology.php.

[24] Miller B, Baig M, Hayes J, Elton S (2006). Injury outcomes in children following automobile, motorcycle, and all-terrain vehicle accidents: an institutional review. J Neurosurg.

[25] Reeping PM, Hemenway D (2020). The association between weather and the number of daily shootings in Chicago (2012-2016). Inj Epidemiol.

[26] Behan DFB (2024). Differences of weekend versus weekdays, nurse-to-patient staffing ratios, patient diagnoses, and direct nursing care time. TWU Coll Nurs.

